# Teachers’ Resources to Support School Lunch: Professional Development Is Warranted

**DOI:** 10.3390/nu14214596

**Published:** 2022-11-01

**Authors:** Deborah A. Olarte, Pamela A. Koch, Randi L. Wolf, Isobel R. Contento

**Affiliations:** 1 Center for Health Inclusion, Research, and Practice (CHIRP), Merrimack College, North Andover, MA 01845, USA; 2 Program in Nutrition, Department of Health and Behavioral Studies, Teachers College, Columbia University, New York, NY 10027, USA

**Keywords:** school lunch, school meals, professional development, school food, school community, K-12 teachers, qualitative research

## Abstract

In the United States, many children who come from low-income backgrounds and experience food insecurity do not take and eat school lunch, despite it being a nutritious meal. Teachers could play a role in encouraging students’ consumption of school lunch; however, teachers in America are traditionally uninvolved in the lunch period. The purpose of this research was to understand the resources kindergarten through twelfth grade (K-12) teachers need to encourage students to take and eat school lunch. Two data collection workshops and semi-structured follow-up interviews were conducted with K-12 teachers. The workshops and interviews were recorded, transcribed, and analyzed for salient themes. Ten teachers participated in the workshops and six teachers participated in the follow-up interviews. In general, teachers believe school meals are essential for students’ focus and behavior in the classroom. However, to encourage students to take and eat school lunch, teachers need support and resources. From the workshops and interviews, three themes emerged: (1) improvements in the food quality; (2) school community support; and (3) professional development. The data suggests professional development is the greatest resource teachers need, as professional development can enhance teachers’ motivation to advocate for better food quality and engage school community support. Greater teacher involvement in school lunch could lay the groundwork for future healthier generations.

## 1. Introduction

Schools are the epicenters of education, making them the ideal setting to improve not only educational, but student health outcomes. Students spend most of the school day with their teachers except for the lunch period [1]. In most schools, teachers eat lunch at the same time as their students but not in the lunchroom as many use that time for lesson planning and prepare for upcoming activities. However, many students do not take and eat school lunch and remain hungry throughout the day for a variety of reasons. This is most problematic in students from a low-income background. Despite having access to free school meals, many experience “in-school food insecurity” while remaining food insecure at home [2]. Teachers could ensure students eat school meals, however, there is minimal research related to teachers supporting school meals, as well as minimal structures in place to assist teachers in this endeavor.

In the United States, lunch is generally not considered an educative opportunity, but a break that students and teachers take during the day. If school lunch education is to be an integral part of the school day, a shift in how school meals are approached must be made. Many students, especially students suffering from food insecurity do not eat at school, out of fear of being stigmatized for getting free lunch [3,4]. Because of this, many kids go hungry during the day and are unfocused in the classroom. The students who benefit the most from school meals are also the students who are most in need of at least one nutritionally sound meal per day [2]. 

A growing body of research has called for food and nutrition education to be connected to school meals in addition to greater teacher involvement [5,6,7]. Teachers are in a unique position in the school setting to promote student health by acting as healthy role models and creating classroom environments where student health efforts are supported [8]. But education does not have to be limited to the classroom and can occur anywhere in the school, including the cafeteria. In 2011, Hightower–Weaver affirmed, “it is time to take school food seriously, to consider how much depends on this most human, embodied part of the school day.” [9]. However, over a decade later, school lunch is still not an educative opportunity and still a separate part of the school day. For students who are financially and nutritionally in need of school meals, this is a missed opportunity. Teachers can make school lunch an educative opportunity and impact student health by playing a greater role in the school lunch served in their schools. Teachers can also encourage students to take and eat school lunch. However, the practices and policies that are in place in most schools minimize teachers’ involvement in school lunch. To change this so that teachers play a greater role, teachers need to build knowledge, skills, and self-efficacy to fully support school lunch. Because school meals can positively impact students’ health and academic outcomes, and students spend most of their day with teachers, research is warranted for teachers be successful in supporting school lunch. Therefore, the purpose of this qualitative study was to understand the resources kindergarten through twelfth grade (K-12) teachers need to play a greater role in encouraging students to take and eat school lunch. 

## 2. Materials and Methods

### 2.1. Research Design

Two data collection workshops followed by semi-structured in-depth interviews relied on a grounded theory approach to gain a deeper understanding of how teachers can realistically support school lunch. In doing so, the participants discussed the resources they need to fully support school lunch.

### 2.2. Recruitment

This study was approved by the Teachers College, Columbia University Institutional Review Board (protocol code 19-124). The participants were recruited via purposive and snowball sampling strategies. To be included, participants had to have at least one year of in-service classroom experience and could attend one of the two, two-hour workshops. Pre-service teachers were excluded. Participants were recruited via flyers and an online portal administered by the hosting academic institution. Recruitment messages were posted daily for two weeks leading up to the workshop. Additionally, snowball sampling was utilized to recruit teachers outside the hosting academic institution. Participants consented to one-hour follow-up interviews after the workshop was completed. 

### 2.3. Data Collection Workshops and Interviews

In both the data collection workshops and the interviews, semi-structured, open-ended questions relating to how teachers could support school lunch were asked. To extract rich qualitative data, the overarching research question guiding the workshops and interviews was: how can teachers realistically support school lunch? Sub-questions highlighted the barriers they face in supporting school lunch, their recommendations and suggestions to support school lunch participation, and the motivations and resources they need to take these actions.

The data collection workshops were conducted in February 2018 and were two hours in length. They were designed to assist teachers in understanding the important role they could play in school lunch and motivate teachers to become advocates for school meals. The workshops were led by the lead researcher and conducted in a comfortable research environment with a robust set of technologies and tools (e.g., set design, audio/visual) that could be configured to support interactive data collection activities and record them orally and visually. The research space was designed by the researcher to extract detailed qualitative data from the teachers. The workshops were divided into three sections designed to encourage teachers to reflect on and discuss school lunch. 

**Figure 1:**
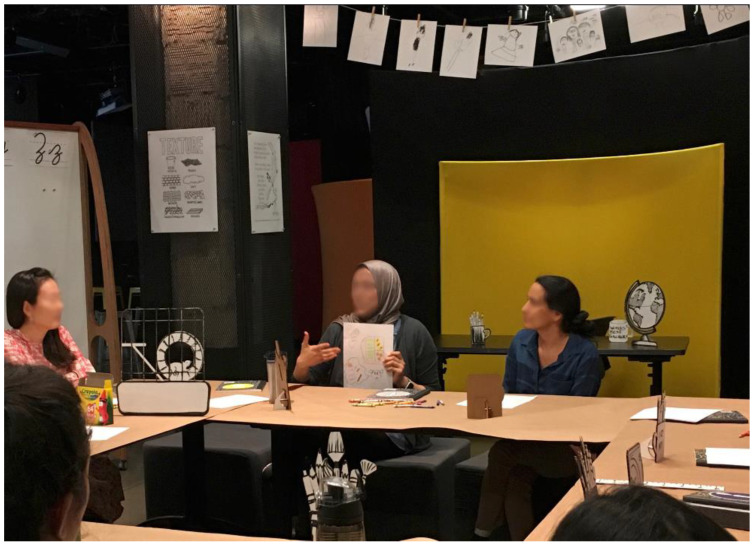
Image from section one of a practice workshop to display the research space and activity. The individuals in the photographs are not the research participants.

In the first section, the teachers were asked to reflect on their childhood school lunch experiences and then asked to draw a memory using paper and crayons. When finished, the teachers were asked to verbally share their drawings and experiences with the group, focusing on how their experiences impacted their current perceptions and practices related to school lunch. Immediately following the first section, the second section explored teachers’ recommendations to encourage greater school lunch participation. The teachers were divided into teams of two or three people and briefed by a video recording from a fictional school principal, explaining a hypothetical new school wellness policy that mandated all teachers speak to their students about the daily lunch served in the cafeteria. Based on this video, the participants were provided a sample menu and were asked to discuss as a team, then deliver to the group three recommendations for how to engage, entice and encourage their students to eat school lunch. These first two sections were used to inspire teachers to think about school lunch and to enable in-depth discussions during the third section. Figure 1 is a photograph from the first section of the workshop displaying the research space and activity.

The third section was a focus group-style semi-structured discussion facilitated by the lead researcher. The data from this focus group was analyzed for this study. Relying on the experiences they had in the first two sections described above, the teachers described the barriers they face in playing a greater role in school lunch. In addition, these discussions aimed to understand teachers’ current and potential future roles in school lunch. Supplementary Materials Figure S1 provides the protocol used for the semi-structured discussions. Because the workshop was two hours in length, a school meal traditionally served in New York City public schools was provided to accompany the groups’ discussion. The intent of the meal was to spark deeper conversation related to school lunch.

The in-depth, individual interviews were conducted between February 2018 and February 2019 at a mutually agreed upon date, time and location between the researcher and the teachers. The interviews allowed the teachers to express themselves in a more intimate setting. The intent of the interviews was to gain a deeper understanding of how teachers could realistically support school lunch and the motivations and resources they need to take these actions. Questions asked include: (1) What are the barriers teachers face in supporting school lunch? (2) What can motivate a teacher to take action to encourage and advocate for school lunch? (3) What are teacher recommendations and suggestions to encourage school lunch participation? (4) What resources are necessary for teachers to support school lunch? 

### 2.4. Data Analysis

The workshops and interviews were audio recorded, transcribed verbatim then checked for accuracy. The transcripts were broadly coded for significant ideas. Then, from thorough reading of the transcripts, a coding scheme was developed. Codes were applied to the data using NVivo, version 12 for Mac [10]. From the codes, themes were created using the coded reports from NVivo. The themes were considered salient based on ideas and discussions with five or more participants. The participants were assigned numbers to maintain their anonymity.

## 3. Results

A total of ten K-12 teachers who taught in schools in and around New York City participated in the workshops. Four participants attended the first workshop, and six participants attended the second workshop. Six participants participated in the follow-up interviews. Among the ten participants, every grade was represented (i.e., at least one teacher taught each grade between kindergarten and twelfth grade at some point in their careers). Out of the ten participants, sixth grade was taught by the most teachers (*n* = 6). Five of the ten participants were between the ages of 21 and 35. Four were between the ages of 36 and 50. One participant did not report their age. Most participants had a graduate degree (*n* = 8). One participant was in the process of obtaining a graduate degree, and one had a doctoral degree. Six participants had been teaching between one and ten years. Four of the participants were Black or African American. Six of the teachers were employed by the New York City Department of Education and the remaining four teachers taught in schools outside of New York City. The teachers participated in this study because they felt school meals were important for students. Of the ten workshop participants, six participated in the follow-up interviews. Table 1 provides teacher background, basic demographic and interview participation information.

From the data, the participants believe school meals are integral to children’s focus and behavior throughout the school day. The participants believed teachers could play a greater role in school lunch through a variety of pathways. However, barriers must first be overcome, and teachers need support and resources to play a greater role in school lunch. For teachers to fully support school lunch, three themes emerged. They were food quality, school community support, and professional development. Ultimately, the participants felt professional development to be the most critical to assist with and enhance achievements with improving food quality and building administrative support.

### 3.1. Food Quality

The participants felt that the quality of the food served in school was a barrier to them supporting school lunch. How the food was presented, and the types of food served to the students were large deterrents for the participants in supporting school lunch. The participants felt it would be hypocritical to positively discuss food they would not eat themselves, as described by the following participant.

Participant 5: There’s no way I would eat that, so it’s just really…I would eat with them but bringing in my own Greek yogurt and granola and strawberries, I don’t know what kind of message that sends. Like, ‘This is okay for you to eat, and you need this’, except I would never tell them they had to, because who am I to say that? I would eat with them, but I would not eat their food.

Many students do not eat school meals for a variety of reasons, and many do not because of taste preferences. Some participants discussed observing students not eating school meals but eating at the local fast-food restaurant before and/or after school. In addition, the participants discussed using food as a reward in their classrooms to feed hungry students who would not eat school lunch. Regarding student hunger during the school day, one participant said,

Participant 9: …they always ask, ‘Are we going to eat something? When are we going to eat?’ And so, I make sure food is a part of everything we do, because it’s such a major issue.

To play a greater role in school lunch, the participants discussed improvements in the quality of the food served at school. If the school lunch was higher quality (i.e., more appetizing scratch-cooked meals and less processed food) they would be willing to participate in school lunch in a larger capacity. The participants also felt they could advocate for improvements to the food served by eating with the students, as described in the following quote.

Participant 8: “I think…getting teachers to sit down and eat it and be like, ‘Oh, this is crap,’ will be an impetus for change.”

Through leading by example, teachers could eat school lunch and role model healthy eating behaviors in the cafeteria, thus encouraging greater student participation. The participants emphasized that eating school lunch should not be a large time commitment or burden for teachers. However, eating school lunch in the cafeteria could also bring students and teachers together and provide time to bond. In addition, eating with the students would allow students to engage with teachers on a personal level through school lunch. This could give teachers greater insight into students’ classroom behaviors after eating lunch with them. as described below.

Participant 4: …dedicate a time either once a week or once a month to eat the same lunch as the students, so that your conversations are a bit more rich. You’re coming from a place of understanding what they’re eating and having the same experience…

### 3.2. School Community Support

Most of the participants felt that they were not capable of making any changes to the food served in the cafeteria. Instead, many blamed their school administrators for the poor-quality food. They felt administrations could do better in procuring higher quality, more appetizing food for students, as described by the following participant.

Participant 7: …or at least, improve the quality of the food that we’re getting for them. But I think that…starts with administration making that decision. And I know that, as far as money, it’s a lot. We are using this company because of X dollars, and then we can use this amount of money to do something else, but really, that money should be put towards the food because that’s really important for nurturing our kids so they can learn.

Lack of administrative support also diminished the motivations teachers would have to be more supportive of school lunch. Without the administration’s buy-in, the participants felt they could not tackle larger initiatives such as supporting school lunch, as described by the participant below.

Participant 10: …you could spend a little bit more money on higher quality food that would then be eaten and not just thrown in the garbage…and they buy filler food. It’s high carbs…it’s just food, empty calories. And if you had something that may have cost more but had more nutrients and everything like that, you would see it would even out. But as a teacher in a school that’s relatively small, you feel like you’re kind of powerless to do anything to help that…I think food is a really big issue and I think it’s something that needs to be changed. But in all honesty, that’s a really uphill battle.

Leadership that provides a top-down approach with systems and procedures would be helpful for teachers to follow. The participants believed plans could only be set in motion by the school’s administration, getting everyone in the school on-board. Additionally, the stresses of class timing and testing is difficult for teachers to manage. However, leadership could alleviate the stress for teachers to work this out on their own. Participants discussed school administrations could be supportive by working out the day-to-day logistics (e.g., scheduling a rotation of teachers to eat school lunch with students, and planning class trips to the cafeteria to meet with food service workers, etc.). Rather than leaving teachers to develop and implement these school lunch connections on their own, the participants felt schools function best when the administration partners with teachers and provides a negotiated plan of action on a daily, weekly, monthly and/or yearly basis.

Participant 2: …there would have to be some kind of plan…A school-wide discussion about how a typical day looks, how a typical week looks, how a typical month looks. How the school year looks. And where that [school lunch education] would fit in across the school year.

The participants felt that as a school community, they all needed to support school lunch together. In addition to their administrators, the participants also agreed that support should come from fellow teachers. Working together and supporting each other to support school lunch was commonly discussed by the participants. For the ease of consistency among teachers, the participants felt it would be easiest for teachers to work and plan together to play a greater role in school lunch.

The participants believed that teachers could play a greater role in school lunch if the school community provided support. Along with teachers, school administrators, food service staff and parents could work together to encourage students to take and eat school lunch. The participants discussed communal events as an opportunity to encourage greater school lunch participation. Communal events ranged from class events, class trips, school-wide events that included parents and competitions between grades. For example, to increase student participation in school lunch, the following quote describes a hypothetical trip for students to meet and interact with the food service staff in the cafeteria.

Participant 3: Maybe having a day where…having the students downstairs talking to the cafeteria staff about what they’re doing with the lunch...and see how they prepare it…just discussing with them how they get all of that prepared…or talking to them about where the food comes from. Does it get delivered? How does it get delivered to you? Do you know what the process is before that?

### 3.3. Professional Development

The participants discussed teachers have much to learn when it comes to school lunch. They felt teachers are generally unaware of the research behind the importance of school lunch for students, nor are they experts on food and nutrition. Some participants expressed apprehension as to whether they’re the right people to educate about food when their personal eating habits may not be ideal. Because of this, the participants suggested that professional development would be helpful to learn how to discuss food with students and positively impact their eating habits. Some participants thought a food and nutrition education course at the university level would also be beneficial for teachers before they begin their careers, as described by the participant below.

Participant 6: I think it would be awesome if teachers had a nutrition-based course. I think everyone needs it. I think it’s huge. I mean, and I also just think understanding food systems is so key to understanding our planet and history and everything else, and it’s just left to the side.

In addition, the participants also mentioned the need for teacher buy-in and felt the way to achieve this would be through professional development. Without teacher buy-in, success at supporting school lunch would be difficult.

Participant 1: I think kids can definitely sense, like with anything else, when there is teacher buy-in. If there is any indication to them that they don’t have teacher buy-in then, if you are not into it, then why should I be into it? I think that’s the biggest thing.

The participants felt teachers could support school lunch and impact student outcomes such classroom focus, behavior and academic performance, if evidence-based professional development was available to elicit teacher buy-in as described in the following quote.

Participant 5: If you can really make the case that research shows that students who eat lunch and eat a balanced lunch…that they’re better behaved and better focused and you’re consistently going to see better outcomes.

To influence teacher buy-in, the participants said it would be helpful to include the importance of school lunch from the perspective that eating school meals can help make students more ready to learn. By teachers playing a greater role in school lunch, their jobs could become easier because their students’ focus and mood will improve. Additionally, teachers would find the most motivation in knowing that food and nutrition are essential tools students need to learn—not just in school, but in life in general as described in the quote below.

Participant 2: …the number one thing is for teachers to understand how important it is for kids to learn about nutrition…being a healthy child and understanding the importance of eating and making good choices around food is really important for learning in a holistic sense.

Through professional development, teachers could improve students’ eating habits and develop knowledge and skills to hold classroom conversations related to school lunch. The participants discussed professional development could also help teachers understand more about the National School Lunch Program (NSLP), and the role it plays in decreasing food insecurity and improving children’s diets. Teachers could also learn skills such as how to encourage greater school lunch participation. However, throughout their careers, the participants had not experienced professional development to assist in supporting school lunch. 

The participants agreed that positively discussing school lunch could help to encourage greater school lunch participation. Additionally, teachers need to believe that school meals are healthy, quality meals. Professional development would help them to positively discuss school lunch with students and have the research to back-up the healthy, quality meals they will be eating. In addition, consistency in the messaging from teachers throughout the school will be beneficial to obtaining student buy-in and greater participation as described below.

Participant 5: Consistency about food across teachers and grade levels is good…because when the students are hearing it from everyone at school, they may be more inclined to eat.

Through professional development, the participants also saw the potential of connecting school lunch to existing curricula to spark discussions. They felt making connections within the curriculum they were already teaching would be an easy way to discuss food and link it to the lunch served in the cafeteria. Connecting the food served at lunch to social studies was an easy way to discuss school lunch. Additionally, English assignments could be an easy way to incorporate school lunch as described below.

Participant 4: …it would be great to tie into writing assignments. I taught English and history. So, I think my first inclination is to do like some sort of debate kind of project or research.

Gardening and cooking were also suggested as ways to connect school lunch to the classroom. Some participants had experience gardening and cooking with students. They discussed growing some of the fruits and vegetables served in the cafeteria, as students could be more willing to eat them if they saw them growing in the school garden. Other participants had experience cooking with their students. One participant was able to cook with ingredients grown in her school garden, as described below.

Participant 6: Our cafeteria director let us cook in the kitchen with her…We would harvest things from the garden, and if it was pizza day, we did a whole series one year where all the pizza slices would have faces made out of herbs and other vegetables that she would bring in... The kids would go to lunch and we would just do this crazy face pizza day or a pasta dish, and she would let us come in and cook.

Regular informal discussions were also an easy means to connect food and nutrition education to school lunch. Informal discussions could include talking with students about the menu on the way to the cafeteria. Reviewing the daily menu at a consistent time every day and encouraging students to try the food was also discussed. 

**Figure 2:**
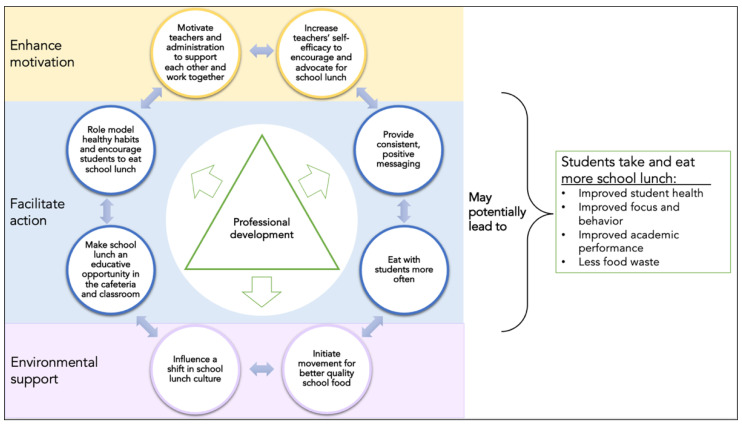
Framework for School Lunch Professional Development based on K-12 teachers’ recommendations and suggestions to play a greater role in school lunch. K-12, kindergarten through twelfth grade.

Data from the workshop and interviews suggest the overall factor that would influence the role teachers can play in school lunch is professional development. Derived from the themes generated from the data, Figure 2 depicts the impact professional development, based on school lunch, can have on teachers and students. 

Professional development can enhance teacher motivation to play a greater role in school lunch, facilitate their actions related to school lunch and help teachers to advocate for, and provide environmental supports. Furthermore, Table 2 provides a listing of quotes supporting Figure 1, illustrating the components of the professional development.

Specifically, professional development is a resource that can enhance teachers’ motivation by raising their awareness about the important role they could play in supporting school lunch and their students’ health and well-being. From the data, learning about the nutritional value school meals provide students and the impact that regularly eating school meals can make on academic performance, teachers could be willing to buy into supporting school lunch. The participants agreed that professional development can also facilitate the actions of teachers by honing their knowledge and skills to role model healthy habits and encourage students to eat school lunch, provide consistent, positive messaging, make school lunch an educative opportunity, and take some time to eat with students. Additionally, professional development would provide teachers with resources that make incorporating more food and nutrition education related to school lunch a burdenless task during the school day. Through professional development, enhancing motivation and facilitating action paves the way for empowerment to advocate for environmental supports to support school lunch and encourage greater school lunch participation.

## 4. Discussion

This study sought to understand the resources teachers need to support school lunch. There are few studies conducted with K-12 classroom teachers related to school lunch in the United States. The data suggest teachers believe school lunch is important for students. However, teachers are overburdened and required curriculum is heavily scheduled throughout the school day. Because many teachers spend most of the school day with their students, they are positioned to provide support to the school lunch program by encouraging students to participate. Yet, for teachers to play a greater role in school meals three resources are warranted. They are improvements in the food quality, school-community support, and professional development. Ultimately, the participants thought professional development was the fundamental necessity to play a larger role in school lunch. 

The participants felt eating school lunch with students would be a way to encourage school lunch participation. Existing research suggests eating school lunch improves students’ diet quality and academic performance [11,12,13,14,15]. At the time of this writing, the Healthy Hunger-Free Kids Act (HHFKA) of 2010 is the most recent federal reauthorization of childhood nutrition policy. The standards required schools to meet childhood caloric needs while increasing the availability of fruits, vegetables, whole grains and low-fat and fat-free milk and reducing levels of sodium, saturated and trans fats in all meals [16]. Despite these improvements, the participants had negative views of school lunch. If they were to encourage greater student participation, they believed improvements to the quality of the meals should be made. Nevertheless, the participants believed that if more teachers began to gradually eat school lunch with students, teachers could collectively advocate for improvements to the meal quality. Role modeling healthy eating habits in the cafeteria could also become a common occurrence because teachers would be choosing to eat school lunch. Additionally, with greater teacher involvement in school lunch, more students will eat school lunch and, their focus and behavior in the classroom will improve. 

The participants believed teachers need a supportive school community to support school lunch. Studies with a school community component have shown success in changing students’ food behaviors as well [17,18]. A supportive school community can be comprised of faculty, administration, food service staff and parents working together to support school lunch. Research shows that a primary approach to improving student health outcomes should include creating healthy school environments [19]. Similarly, research has shown that a decrease in support of a healthy school environment correlates with little motivation for teachers to role model healthy behaviors in the classroom [20]. Without the buy-in of the school administrators the participants felt that supporting school lunch would be difficult. Four studies similarly noted administrative support to be essential for program success [21,22,23,24]. Therefore, if administrators assist teachers in supporting school lunch, more students could be influenced to take and eat school lunch. To assist teachers with day-to-day scheduling, administrators could help by providing specific yearly, monthly and weekly goals and plans and provide school lunch menus to teachers. Parents and food service staff can become more involved through class and school community events.

Ultimately, the participants discussed professional development as central to their role in school lunch. Professional development can provide teachers with motivation for why to support school lunch, simple methods for how to support school lunch and the methods and motivation to advocate for improvements to school lunch. Research has called for more teacher professional development related to school meals and feeding strategies [25,26,27]. Research has also shown professional development to be beneficial in improving students eating habits in pre-school children [28,29,30]. With professional development focused on motivating teachers, facilitating action and improving environmental supports, teachers could be successful at improving students’ focus, behavior, academic performance and health [31].

In 2015, the Whole School, Whole Community, Whole Child (WSCC) model was developed by the Association for Supervisors and Curriculum Development and the Centers for Disease Control [26]. The WSCC calls upon higher education institutions to design coursework, preservice and in-service events, and professional development for teachers and others, directly and indirectly involved with schools to help implement the model [26]. Along with professional development, food and nutrition education for preservice teachers was discussed by the participants and can provide teachers with a foundational understanding of the food system, school food policy federal programs to support child hunger, and childhood nutrition. Calling on higher education to provide course work and other activities to preservice teachers on food, nutrition, and school lunch has also been seen in previous literature [32,33]. Through a food and nutrition education course for educators, preservice teachers can build knowledge, skills and self-efficacy to discuss school lunch with students. Discussions could range from the environment to culture to social justice [6]. They can reflect on their own school lunch experiences and understand how those experiences could impact their students [34]. Future teachers could also be inspired to incorporate school lunch, cooking and gardening with students into their curriculum [6]. 

Around the world, there are school lunch programs where teachers play a large role. The National Food Administration (NFA) in Sweden publishes guidelines for school meals served from preschool through high school [35]. Though not mandated, these guidelines strongly recommend a “pedagogic meal” to provide children the chance to interact with teachers outside of the classroom and teach children about food, nutrition and healthy eating. The NFA believes the presence of an adult in the cafeteria makes the meal environment less chaotic and recommends teachers regularly eat with the students and have positive discussions about the school lunch [35].

A 2017 study conducted in Swedish schools explored the extent teachers eat school lunch with their students in the cafeteria, their attitudes toward the success of the pedagogic meals and whether teachers see themselves as school lunch role models [36]. The majority of teachers (90%) ate lunch with their students more than one day per week while 51% ate with the students every day. Seventy-two percent felt school lunch should be an educative opportunity where issues of food waste and healthy eating could be discussed. Because they ate with the students, the majority of teachers questioned also felt they were school lunch role models. It should be pointed out that the teachers in Sweden are not required to eat with the students, yet it is part of their culture and beliefs that school lunch is important for students’ learning [36].

Similarly, In Finland, school meals and food education are an essential part of the school day. Guided by the Finnish National Nutrition Council’s School Meal Recommendations, students receive free school meals and teachers regularly eat with the students [37]. To assist teachers in integrating school lunch into the school day and create an overall healthy school environment, Finland implemented the “Tasty School” model that integrates food and nutrition education into curriculum, school meals and the school environment. “Tasty School” is teacher-led with tools and resources provided to teachers to support the model. A recent study evaluated teachers’ perceptions of, and experiences with food and nutrition education and school meals, as well as the feasibility of implementing the teacher-led “Tasty School” model. Results show positive teacher responses toward the new model. The teachers felt “Tasty School” put food and nutrition education into practice and benefitted the school’s teaching. Additionally, the teachers reported principals to be a critical facilitator in implementing the model and providing effective food and nutrition education [37]. 

In Japan, there is the Law for School Lunches, or the Gakkou Kyushoku-hou, that mandates school lunch be an educative experience and not a break in the day [38]. Japan has a unique school lunch experience where teachers play a direct role with school lunch because takes place in the classroom. The food is prepared daily, and students play an active role in the set-up and clean-up of the meal. The students enjoy the meal and on average only 6.9% of food is wasted, according to a survey on food loss by the Japanese Ministry of the Environment in 2014 [39]. Because the meal takes place in the classroom, teachers eat with their students and provide lessons in etiquette, food, ecology and other subjects [38]. 

However, in the United States, academic pressure is a barrier to supporting school lunch [24,27,40]. Teachers are overburdened and required curriculum and testing preparation is tightly scheduled throughout the school year. No Child Left Behind, passed in 2002, implemented nationwide standardized testing, thought to be an ideal, objective measurement of educational status across the United States [41]. However, standardized test scores assume the quality of the education and do not account for societal and environmental factors, such as hunger, that may impact students’ learning in the classroom. Despite assistance from school administrators, when testing is a priority, teachers will have a difficult time prioritizing food and nutrition education and school meals. To create an overall healthy school environment, federal and state education policies could begin prioritizing student health and nutrition over standardized testing [6].

Simultaneously, schools may want to take a school-wide approach in modifying how school lunch is portrayed and discussed. Schools could also structure school lunch programs to encourage all students to partake in lunch and, more importantly, not to stigmatize food insecure students. This is particularly important in schools with few food insecure students, as these students may be more aware that they are different [7,9]. Perhaps the initiation of a federally mandated universal free school meals program (UFSM) is the key to decreasing the socioeconomic stigma of school lunch and increase participation [42]. A recent systematic review examined the associations between a UFSM program and participation rates, diets, attendance, academic performance, and Body Mass Index (BMI), as well as school finances [43]. From the research, a UFSM program increases student participation and is positively associated with diet quality, attendance and academic performance. In addition, a free school meals program may lower students’ BMI levels. Lower income schools may also see positive financial outcomes when more students participate. Because stigma is among the main reasons students do not participate in school meals, teachers could advocate for a UFSM program in the United States [3,4]. A UFSM program with teacher involvement and an informal school lunch curriculum could thereby lay the groundwork for a healthier generation.

This study has several strengths and limitations. This is the first study to focus on the needs of teachers to support school lunch. The workshops took place in a space specifically for interactive workshops and the two data collection workshops were slotted into specific dates and times. Therefore, the potential for including a greater number of participants was not possible. The sample of participants were a total of ten. Because of this small sample, data saturation may not have been reached. However, research suggests up to 80% of themes are discoverable after two focus groups and saturation can be reached after six interviews [44,45]. Additionally, these limitations were minimized by conducting follow-up interviews to gain more perspective. 

The interviews gave participants the opportunity provide additional information in a more intimate setting. Additionally, the qualitative methods used in both the workshops and interviews extracted rich qualitative data. The teachers who participated came to the workshop because they had a prior interest in food and nutrition. Therefore, the participants’ responses may have been biased in a positive direction. There was a lack of diversity in the physical location of the teachers as many lived and/or worked in a large metropolitan area. However, all teachers were public school teachers with similar experiences. Four of the ten teachers that were employed by other school districts had more flexibility in where they ate lunch and could add a different perspective with their experiences related to school lunch. Similarly, teachers in other areas of the United States may have other views and experiences related to school lunch. Additionally, between the ten participants, all grades between kindergarten and twelve were represented. Finally, relying on a grounded theory approach, this research lays the groundwork for teachers to play a greater role in school lunch and provides the framework for a school lunch-based professional development that can be put into practice by teachers. 

## 5. Conclusions

This study suggests a variety of ways, specifically resources K-12 teachers need to better support and encourage students to take and eat school lunch. Specifically, a school lunch-based professional development for teachers is warranted. Future quantitative research could evaluate the effectiveness of professional development by examining teachers’ pre- and post-knowledge and self-efficacy related to school lunch. Cafeteria observations could also be made to compare differences pre- and post-professional development. Additional research can assess students’ health, dietary intake and academic performance after teachers play a larger role in school lunch. The research also suggests the creation and implementation of university-level courses that would provide pre-service teachers the opportunity to be educated in nutrition. This would provide teachers entering the workforce with the knowledge and self-efficacy they will need to successfully implement and deliver food and nutrition education related to school lunch in their classrooms.

## Figures and Tables

**Table 1:** Participant Background, Basic Demographic and Interview Information.

Participant	Age Range	Degree Earned	Years Teaching	Grade(s) Taught	Subject(s) Taught	Race and Ethnicity	Interview Participant
1	36–50	Graduate	11–20	7–12	History	Other, Non- Hispanic	N
2	36–50	Doctorate	11–20	1	General Education	White, Non- Hispanic	Y
3	21–35	Graduate	1–10	8	Music	African American, Non-Hispanic	Y
4	-	Graduate	1–10	3, 6, 7, 8	General Education	White, Non- Hispanic	N
5	21–35	Undergraduate	1–10	6	Math	White, Non- Hispanic	Y
6	21–35	Graduate	1–10	6	English	White, Non- Hispanic	Y
7	21–35	Graduate	1–10	K-12	Physical Education	African American, Non-Hispanic	Y
8	21–35	Graduate	1–10	6–12	-	African American, Non-Hispanic	N
9	36–50	Graduate	21–30	6–12	Health	African American, Non-Hispanic	N
10	36–50	Graduate	11–20	K-8	Science	White, Non- Hispanic	Y

-, not provided.

**Table 2:** Illustrative quotes from participants related to the components of professional development (i.e., enhancing motivation, facilitating action, and environmental supports).

**Participant**	**Quotes Related to Enhancing Motivation**
6	“Maybe just hearing from other teachers of their successes. I think teachers are really motivated by seeing examples of something they want to emulate…if there was a prize…if there were those kinds of things, like, ‘Hey, we want to highlight a school with the best cafeteria practices,’ and then some teachers get acknowledged and they get published in a UFT paper. And everyone’s like, ‘Ooh, I want to be that teacher.”
7	“…I definitely think having the teachers band together to advocate in a way of, we are seeing this in our classroom. And we also think that this solution could help…and have an actual plan.”
**Participant**	**Quotes related to facilitating action**
3	“Maybe…having the students downstairs talking to the cafeteria staff about what they’re doing with the lunch...and see how they prepare it…just discussing with them how they get all of that prepared…or talking to them about where the food comes from.”
4	“…dedicate a time either once a week or once a month to eat the same lunch as the students, so that your conversations are a bit more rich. You’re coming from a place of understanding what they’re eating and having the same experience…”
8	“…I decided I am going to start a club where we are just going to talk about food, nutrition…And so the way that I structured it was just like, a space to talk about the way they interact with food at home, and what their challenges were, what their experiences were with food.”
10	“I decided I’m just going to grow herbs in it because they’re easy. They’re fast growing. And I could do basil and parsley and I could do something easy. We can make pizza, put the basil on the pizza. We could do parsley, make the pesto and different herbs to do something light...”
**Participant**	**Quotes related to environmental support**
4	“…but it’s something in terms of creating an environment where not only is the food better, healthier and consciously chosen, but the environment is one which is like, ‘okay, I can catch my breath from a morning of learning and like talk with my friends...”
6	“I do think you need someone from the top down coming in and saying, ‘Here’s our systems. Here’s out procedures. And hey teachers, there’s a really clear way that this works and it’s manageable…”
**Participant**	**Quotes related to professional development**
3	“Some kind of development of, this is what school lunches normally look like. Even, these are the amount of your students who actually eat the school lunch. These are the things that we know happen when students do eat lunch versus don’t eat lunch. And stuff like that where the teachers can get engaged in.”
2	“Good professional development around nutrition education. Just presenting the idea at all that this is an important thing to teach in schools. That children need to learn it.”
7	“I think there should be so many more courses in teacher education programs, and one of them should be nutrition... I have so many... One should be actual teacher professional socialization, and what it really is like when you get in the classroom…But I agree, I think a health education or nutrition education course should be, or health education, should be involved with a social justice lens.”

UFT, United Federation of Teachers.

## Data Availability

Not applicable.

## Supplementary Materials

The following supporting information can be downloaded at: https://www.mdpi.com/article/10.3390/nu14214596/s1, Figure S1: Semi-structured focus group-style discussion protocol that guided section three of the workshop with the teachers.
