# Supplementary material for: Teachers’ Resources to Support School Lunch: Professional Development Is Warranted
Source: Nutrients. 2022 Nov 1;14(21):4596. doi: 10.3390/nu14214596 (PMC9655880; doi:10.3390/nu14214596)
Supplement: Supplementary file 1 [file nutrients-14-04596-s001.zip › nutrients-1941484-supplementary.pdf]

The Focus Group
**Question 1: Why do teachers support, ignore or condemn school lunch?**<br><br>Sub-question 1: Do you believe school lunch can improve academic performance, and if so, why?<br><br>Sub-question 2: What are your thoughts on handling encounters with students who are eating competing foods (i.e. food from a vending machine or corner store)? Would you help them to try a healthier option? If so, how? If not, why?<br><br>Sub-question 3: What do school administrators do to help or hinder your supporting school lunch?<br><br>Sub-question 4: What are some ways teachers can be better advocates for school lunch?<br><br>Sub-question 5: Despite logistical reasons for not participating more in school lunch (i.e. union rules) what are some other reasons teachers don't engage with the lunch period?
**Question 2: What are teachers current practices related to school lunch?**<br><br>Sub-question 1: Do you feel you have a role to play in your student's health? Why, or why not? If so, what is that role? What were the differences before versus after the workshop?<br><br>Sub-question 1: What are your beliefs and practices regarding food and nutrition education and school lunch? What is different after participating the workshop?<br><br>Sub-question 2: How do you participate in your school's lunch program? What will you do now, after the workshop?<br><br>Sub-question 3: Do you do anything in your classroom related to food and nutrition education? If so, what do you do? What can you do now, after participating in the workshop?<br><br>Sub-question 4: Do you do anything to link food and nutrition education to your school's lunch? If so, how? What are some ideas to link food and nutrition education and school lunch, now having participated in the workshop?<br><br>Sub-question 5: How do you think greater teacher involvement in school lunch would help improve students eating habits at school? And if not, why? Do you think this workshop will help improve teacher involvement in student eating habits?
Focus group leader: This completes the questions I have for you today. Are there any topics discussed in which you would like to elaborate on further? Is there anything else you would like to add that I have not addressed today?

Figure S1. Semi-structured focus group-style discussion protocol that guided section three of the workshop with the teachers.
